# The Free and Cued Selective Reminding Test in Parkinson's Disease Mild Cognitive Impairment: Discriminative Accuracy and Neural Correlates

**DOI:** 10.3389/fneur.2020.00240

**Published:** 2020-04-21

**Authors:** Andrea Horta-Barba, Javier Pagonabarraga, Saül Martínez-Horta, Juan Marín-Lahoz, Frederic Sampedro, Ramón Fernández-Bobadilla, M. Ángeles Botí, Helena Bejr-Kasem, Ignacio Aracil-Bolaños, Jesus Pérez-Pérez, Berta Pascual-Sedano, Antonia Campolongo, Cristina Izquierdo, Beatriz Gómez-Ansón, Jaime Kulisevsky

**Affiliations:** ^1^Movement Disorders Unit, Neurology Department, Sant Pau Hospital, Barcelona, Spain; ^2^Biomedical Research Institute (IIB-Sant Pau), Barcelona, Spain; ^3^Centro de Investigación en Red-Enfermedades Neurodegenerativas (CIBERNED), Barcelona, Spain; ^4^Autonomous University of Barcelona, Barcelona, Spain; ^5^Neuroradiology Unit, Sant Pau Hospital, Barcelona, Spain

**Keywords:** PD-MCI, Free and Cued Selective Reminding Rest, Parkinson's disease, memory, episodic memory

## Abstract

**Introduction:** Memory alterations are common in Parkinson's disease (PD) patients but the mechanisms involved in these deficits remain poorly understood. The study aims to explore the profile of episodic memory deficits in non-demented early PD patients.

**Methods:** We obtained neurological, cognitive and behavioral data from 114 PD patients and 41 healthy controls (HC). PD participants were grouped as normal cognition (PD-NC) and mild cognitive impairment (PD-MCI) according to the Level II criteria of the Movement Disorders Society Task Force (MDS-TF). We evaluate the performance amongst groups on an episodic memory task using the Free and Cued Selective Reminding Test (FCSRT). Additionally, gray matter volume (GMV) voxel based morphometry, and mean diffusivity (MD) analyses were conducted in a subset of patients to explore the structural brain correlates of FCSRT performance.

**Results:** Performance on all subscores of the FCSRT was significantly worse in PD-MCI than in PD-NC and HC. Delayed total recall (DTR) subscore was the best at differentiating PD-NC from PD-MCI. Using crosstabulation, DTR allowed identification of PD-MCI patients with an accuracy of 80%. Delayed free and cued recall was associated with decreased GMV and increased MD in multiple fronto-temporal and parietal areas.

**Conclusion:** Encoding and retrieval deficits are a main characteristic of PD-MCI and are associated with structural damage in temporal, parietal and prefrontal areas.

## Introduction

At the time Parkinson's disease (PD) diagnosis, up to 30% of patients meet diagnostic criteria for mild cognitive impairment (PD-MCI) (1–3). The rate of progression of PD-MCI is heterogeneous, with up to 36% of patients fulfilling diagnostic criteria for PD dementia (PDD) after 4 years after diagnosis, with cumulative PDD prevalence of 80% in 20 years long-term survivors (4–6). Thus, despite dementia is not an inevitable consequence of PD, it affects a significant proportion of patients for which treatments to ameliorate this entity are lacking.

Identifying early cognitive indicators suggestive of progression to dementia is a major need to stratify patients in different groups of risk and also to design interventions before PDD onset. In this sense, the addition of posterior-cortical type deficits -to the prototypical frontal-executive alterations seen in most PD patients- seem to characterize the transition from PD-MCI to PDD in this population. Accordingly, the development of language, memory and visuospatial/visuoperceptive alterations are indicative of a more aggressive progression of cognitive deterioration in PD (7, 8).

Episodic memory alterations are also found in PD and affects up to 45% of *de novo* PD patients. However, its role in the delineation of progression from PD-MCI to PDD and the mechanisms participating in these deficits has been scarcely studied (9). Attention and retrieval deficits -rather than storage and retention alterations- has been pointed to sub-serve episodic memory difficulties in PD (10). This is supported by the benefit commonly observed in retrieval when semantic or recognition cues are presented to PD patients. Accordingly, decreased performance in memory tasks in PD have been attributed to frontal-executive deficits rather than to hippocampal or medial temporal lobe alterations. However, difficulties in retrieving information even during recognition and cued-facilitated recall have also been described in PD (11). This suggests that in some patients amnestic difficulties may be associated to hippocampal alterations rather than been restricted to frontal-executive alterations. The role that this kind of deficits might play in PD-MCI and in the conversion to PDD is mostly unknown. However, exploring differences in episodic memory performance in non-demented PD patients with and without PD-MCI may help to delineate early cognitive changes with significant prognostic implications in terms of cognitive progression.

In the present study, we aimed to explore the profile of episodic memory deficits in non-demented early PD patients with normal cognition (PD-NC) and PD-MCI. To explore the extent of structural brain differences accompanying these deficits, we also conducted voxel based morphometry (VBM) and mean diffusivity (MD) analyses in a subset of participants. Gray matter volume (GMV) analyses through VBM is a macrostructural neuroimaging technique that has been widely used to characterize brain atrophy. In recent years, increases in MD both in white-matter and gray-matter tissues have been suggested to infer microstructural brain damage.

## Methods

### Participants

We prospectively recruited 114 PD patients who fulfilled the UK Brain Bank Diagnostic Criteria for PD and regularly attending the Movement Disorders Unit at our center and a group of 41 age-matched and education-matched healthy controls. The study procedures included a neurological examination and the administration of a comprehensive neuropsychological assessment battery which was done to all participants, including patients and healthy controls. Presence of PDD according to consensus guidelines (12); having undergone deep brain stimulation surgery; brain abnormalities evidenced in imaging studies performed in the previous year; major depression; treatment with anticholinergic drugs; and any known causes of cognitive impairment other than PD defined exclusion criteria. Written informed consent was obtained from all participants and all procedures were performed in accordance with the standards of the local Ethic Review Board of the Sant Pau hospital in Barcelona, and with the 1964 Helsinki declaration and its later amendments.

### Procedures

Data was collected during two separate visits. Data at screening included: age, educational level, current medications with dopaminergic drugs converted to levodopa equivalent daily dose (LEDD), formal application of the MDS criteria to exclude PDD, the MDS-Unified Parkinson's Disease Rating Scale part III (UPDRS-III) motor subscale, Hoehn, and Yahr (H&Y), and the Parkinson's Disease-Cognitive Rating Scale (PD-CRS), which is a screening instrument that addresses global cognition. On the second visit, a comprehensive neuropsychological examination that fulfilled the standards proposed by the MDS Task Force for the diagnosis of PD-MCI was completed (2).

#### Neuropsychological Assessment and Group Classification

PD participants were grouped as PD-NC and PD-MCI according to the Level II criteria of the Movement Disorders Society Task Force (MDS-TF) for the diagnosis of PD-MCI (1, 2). Thus, five cognitive domains (attention, language, memory, visuospatial skills, and executive functions) were examined using a comprehensive battery composed of two tests per domain. We applied cut-offs of 1.5 SD below normative values and PD-MCI was confirmed when any two (or more) impaired neuropsychological test were present (2, 13). The following standardized and recommended neuropsychological measures were used: Parkinson's Cognitive dementia rating scale (PD-CRS), forward and backward Corsi's block-tapping task, forward digit span task, phonetic and semantic verbal fluency, the Rey-Osterrieth complex figure test, the Boston Naming test, the Judgment of Line Orientation, and the number location subtest of the Visual Object and Space Perception Battery.

Episodic memory was assessed using the Free and Cued Selective Reminding Test (FCSRT). The FCSRT is a widely used episodic memory test which assesses immediate and delayed free-recall and cued-facilitated immediate and delayed recall. In studies assessing memory performance in MCI in the general population, the FCSRT has shown to reflect hippocampal-mediated consolidation memory defects better than free-list learning tests. Moreover, the FCSRT performance predicts progression to dementia in close relationship with progressive atrophy of the medial temporal lobe and other neocortical temporal and parietal regions (10, 14–19). The FCSRT was administered using standard procedures as described by Grober and Buschke (20). Participants were shown a card with four words, and were asked to determine which one of the four corresponds to a particular category (e.g., cue; clothing, and the word “vest”). The participant should learn the four items on the four cards (total 16 words). Three recall trials were conducted, each one preceded by 20 s of counting backwards used as interference. For each trial, participants were asked to freely recall as many items as possible and category cues were provided for items not retrieved by total free recall. The same procedure of recalling (freely and cued) was done after a 30 min interval. Subjects were required to freely remember the words and category cues were provided for items not retrieved freely. The measures evaluated here were: total free recall-TFR (cumulative sum of free recall from the three trials; range 0–48), total recall-TR (cumulative sum of free recall + cued recall from the three trails, range 0–48), delayed free recall-DFR (free delayed recall, range 0–16), and delayed total recall-DTR (free delayed recall + cued delayed recall, range 0–16).

Both patients and healthy controls followed the same assessment.

#### Neuroimaging Acquisition

A subsample of 56 patients underwent 3-Tescla Magnetic Resonance Imaging (MRI) (Philips Achieva). T1-weighted MRI acquisition was performed using a dedicated axial T13D-MPRAGE MRI (TR/TE, 500/50 ms; flip angle, 8, field of view [FOV], 23 cm; with in-plane resolution of 256 × 256 and 1-mm slice thickness). Diffusion Tensor Imaging (DTI) scans were also obtained (FOV 220 mm, voxel size 2 mm, TR 8,000 ms, TE 80 ms, flip angle 90°, 32 directions, b-factor 1000). The neuropsychological and MRI scans were performed within a maximum of 3 months between procedures.

### Statistical Analysis

Data are expressed as means ± standard deviation (SD) for the continuous variables and as mean range for the ordinal variables. Differences between groups were analyzed with independent two-tailed *t*-tests and analyses of variance (ANOVA) for continuous variables, the Mann-Whitney test for ordinal data, and the χ2 test for categorical variables. Comparison of clinical, demographic, and neuropsychological data between groups were done using One-Way ANOVA between the three groups, with additional Tukey *post-hoc* tests for more direct comparisons between each pair of groups.

Binary logistic regression analysis was performed to test the independent classification capacity of the different FCSRT subscores.To calculate the effect size of the differences observed between cognitive groups we used Cohen's *d* coefficient (d values: 0–0.3, small effect size; 0.3–0.6, moderate effect size; >0.6, large effect size). Receiver operator characteristic (ROC) curves were generated to explore the discriminative capacity of each FCSRT subscore. We used cross-tabulation to calculate diagnostic accuracy. Associations between demographic, clinical and cognitive variables were studied using Pearson's correlations. Significance was set at *p* < 0.05. All the statistical procedures were performed using the SPSS v16.0 statistical software package.

#### Neuroimaging Analysis

A voxel-based morphometry (VBM) analysis of gray matter volume (GMV) was performed using the Statistical Parametrical Mapping (SPM12) software (http://www.fil.ion.ucl.ac.uk/spm/). T1-MRI images were segmented to obtain GMV probability maps, which were then normalized to the Montreal Neurological Institute (MNI) stereotactic space using DARTEL. The resulting images were smoothed using a Gaussian kernel of 8 mm full width at half maximum (FWHM).

DTI images were preprocessed using FSL 5.0 software (https://fsl.fmrib.ox.ac.uk/fsl/fslwiki). First, non-brain tissue was removed using the Brain Extractor Tool (BET). Second, motion and eddy current correction was performed using the FMRIB's Diffusion Toolbox (FDT). Diffusion tensors were then computed and mean diffusivity (MD) maps were obtained for each patient. These maps were then normalized to MNI space and smoothed using an isotropic filter of 6 mm FWHM.

The normalized GMV and MD images were entered into a voxel-wise multiple regression analysis to explore the brain correlates of the DFR and DTR scores in both modalities. Age, sex, education and total intracranial volume were used as covariates of no interest. Voxelwise imaging results showing *p* < 0.005 (uncorrected) and a minimum cluster extent size of *k* = 100 voxels was considered significant (21–23). Clusters surviving family-wise error (FWE) correction for multiple comparisons are reported in the corresponding cluster description table (**Table 4**). The MRIcron software tool (https://www.nitrc.org/projects/mricron) was used to represent the statistical voxelwise maps.

## Results

One hundred and fourteen PD patients (68.0 ± 8.3 years) and 41 healthy controls (HC; 66.3 ± 7.4 years) were included in the study. PD patients were in the early to mid-stages of the disease (disease duration 5.3 ± 3 years; H&Y stage 2.0 ± 0.2; UPDRS-III 25.2 ± 8.1). As seen in Table 1, PD patients and HC were matched for age, gender and education.

**Table 1:** Clinic and sociodemographic characteristics of the sample.

	**Controls**	**PD total sample**	**PD-NC**	**PD-MCI**	P^*^	***P*^†^**
Age (years)	66.3 ± 7.4	68 ± 8.3	67.5 ± 8.1	72.9 ± 4.9	0.251	0.012
Gender (m/f)	21/20	72/42	64/28	8/14	χ^2^ = 0.125	χ^2^ = 0.004
Education (years)	13.2 ± 4.8	12.2 ± 4.7	13 ± 4.4	8.7 ± 4.4	0.250	<0.001
Disease duration (years)	–	5.3 ± 3.4	5 ± 3.1	6.1 ± 4.2	–	0.278
MDS-UPDRS III^a^	1.2 ± 1.2	25.2 ± 8.1	24 ± 7.4	29.8 ± 9	<0.001	<0.01
H&Y^b^	–	2 ± 0.2	2 ± 0.2	2 ± 0.2	–	0.891
Total LEDD^c^	–	565 ± 312	571 ± 320	540 ± 282	–	0.680

a *Movement Disorders Society—Unified Parkinson's Disease Rating Scale part III*.

b *Hoehn and Yahr stage*.

c *Total levodopa equivalent daily dose*.

* *P-values were determined with t-test for independent samples between healthy controls and PD*.

† *P-values were determined with t-test for independent samples between PD-NC, and PD-MCI*.

According to MDS-TF level II criteria for PD-MCI, using a detection threshold of −1.5 SD, 22 of the 114 patients (19.0%) were classified as having PD-MCI and all HC were classified in the range of normal cognition, following this same criteria. Looking at the different cognitive groups, those in the PD-MCI group were significantly older than PD-NC [*t*_(113)_ = 3.174; *p* < 0.01] and HC [*t*_(63)_ = 3.78; *p* < 0.01], have lower educational level than PD-NC [*t*_(113)_ = −4.18; *p* < 0.01] and HC [*t*_(63)_ = −3.66; *p* < 0.01], and had higher UPDRS-III score than PD-NC [*t*_(63)_ = 3.10; *p* < 0.05].

As depicted in Table 2, between-group comparisons showed significant differences in all the cognitive measures with the exception of the number location subtest of the VOSP. *Post-hoc* comparisons showed no significant differences in cognitive performance between PD-NC and controls. Conversely, PD-MCI performed significantly worse than PD-NC in all cognitive measures with the exception of the number location subtest of the VOSP.

**Table 2:** Level I and Level II assessment scores.

	**Controls^a^**	**PD-NC^b^**	**PD-MCI^c^**	***ANOVA***	***Turkey's***
PD-CRS Total score	101.2 ± 12.9	95 ± 15.4	73 ± 7.1	<0.001	^a−b^0.056; ^b−c^ <0.001
Frontal-subcortical	72.3 ± 12.1	66.4 ± 14.6	44.8 ± 7	<0.001	^a−b^0.057; ^b−c^ <0.001
Posterior-cortical	28.9 ± 1.7	28.5 ± 1.7	27.1 ± 2	<0.001	^a−b^0.594; ^b−c^ <0.005
**Attention**					
Corsi Forward	5.2 ± 0.9	5.2 ± 0.9	4.1 ± 1	<0.001	^a−b^0.987; ^b−c^ <0.001
Digit span forward	5.5 ± 1	5.5 ± 1.1	4.6 ± 0.7	<0.01	^a−b^0.947; ^b−c^ <0.005
**Executive functions**					
Phonetic fluency	15.4 ± 4.6	15.5 ± 4.9	8.7 ± 3.4	<0.001	^a−b^0.993; ^b−c^ <0.001
Corsi Backward	4.8 ± 0.9	4.7 ± 1	3.4 ± 0.9	<0.001	^a−b^0.878; ^b−c^ <0.001
**Memory**					
PD-CRS delayed memory total recall	6.6 ± 1.8	6.2 ± 2.4	4.5 ±1.7	<0.001	^a−b^0.660; ^b−c^ <0.001
ROCFT −30 min^1^	16.1 ± 5.8	13.3 ± 6.8	4.8 ± 5.3	<0.001	^a−b^0.076; ^b−c^ <0.001
**Language**					
BNT-60^2^	54.6 ± 6.7	54.7 ± 4.6	47.7 ± 5.2	<0.001	^a−b^0.685; ^b−c^ <0.001
Semantic fluency	20.6 ± 4.8	19 ± 5.5	12.1 ± 3.2	<0.001	^a−b^0.229; ^b−c^0.001
**Visuospatial**					
JLOT^3^	23.3 ± 4.6	22.4 ± 5	15.1 ± 6.9	<0.001	^a−b^0.679; ^b−c^ < 0.001
VOSP—number location^4^	19.7 ± 1	19.6 ± 0.9	19.3 ± 1.2	0.351	^a−b^0.907; ^b−c^0.424

1 *Rey-Osterrieth complex figure test −30 min delayed recall*.

2 *Boston Naming Test −60 items*.

3 *Judgement of line orientation test*.

4 *Visual object and shape perception test*.

a−b *Controls vs. PD-NC*.

b−c *PD-NC vs. PD-MCI*.

Looking at the FCSRT test performance, no differences were found between PD-NC and HC in any of the obtained FCSRT subscores. Conversely, performance of the patients in the PD-MCI group was significantly worse than performance of the HC and the PD-NC in all the subscores (*p* < 0.001). As reflected by Cohen's *d*, large effect sizes were found for TFR (*d* = 1.09), TR (*d* =1.23), DFR (*d* = 1.03), and DTR (*d* = 1.60) when comparing PD-MCI with PD-NC. Large effects were also found when comparing PD-MCI with HC in all FCSRT subscores: TFR (*d* = 1.31), TR (*d* = 0.94), DFR (*d* = 1.05), and DTR (*d* = 1.14). When comparing PD-NC with HC with Cohen's *d*, we found small effects in all FCSRT subscores: TFR (*d* = 0.29); TR (*d* = 0.11); DFR (*d* = 0.06); and DTR (*d* = 0.10). We used stepwise logistic regression analysis (forward; conditional) to determine FCSRT subscores that independently differentiated PD-NC from PD-MCI. The variables found to be significantly different between cognitive groups in the one-way ANOVA were included in the analysis to assess their contribution to group discrimination. From all the variables included in the model, the DTR (*r* = 549; *p* < 0.001) was the best differentiating PD-NC from PD-MCI independently on age, education or UPDRS-III score (see Table 3).

**Table 3:** Comparative FCSRT performance between PD-MCI, PD-NC and HC.

	**Controls^a^**	**PD-NC^b^**	**PD-MCI^c^**	***ANOVA***	***Turkey's***	Cohen's d^*^
**FCSRT**						
Total free recall	26.7 ± 7.5	24.5 ± 7	16.7 ± 7	<0.001	^a−b^0.300; ^b−c^ <0.001	1.09
Total recall	41.6 ± 6.7	42.8 ± 5.1	35.1 ± 9.1	<0.001	^a−b^0.872; ^b−c^ <0.001	1.23
Delayed free recall	9.6 ± 3.1	9.4 ± 3	6.4 ± 3	<0.001	^a−b^0.952; ^b−c^ <0.001	1.03
Delayed total recall	14.2 ± 2.6	14.4 ± 1.8	11 ± 3	<0.001	^a−b^0.878; ^b−c^ <0.001	1.60

a−b *Controls vs. PD-NC*.

b−c *PD-NC vs. PD-MCI*.

* *Cohen's d for PD-NC vs. PD-MCI comparisons*.

### Discriminative Capacity of the FCSRT

**Figure 1:**
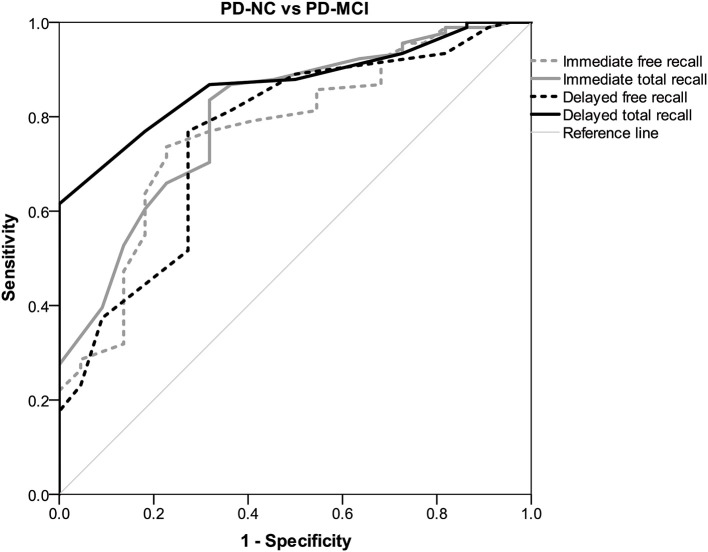
Receiver operating characteristic (ROC) curves illustrating the discriminative properties of the FCSRT.

Receiver operating characteristics (ROC) curves to discriminate between PD-MCI and PD-NC indicated that a cut-off of ≤ 20/21 points on the TFR score yielded sensitivity, 73%; specificity, 77% (AUC = 0.770; 95% confidence interval, 0.663–0.878, *p* < 0.005). A cut-off score ≤ 38/39 points on the TR score showed sensitivity, 80%; specificity, 69% (AUC = 0.803; 95% confidence interval, 0.704–0.902, *p* < 0.001). A cut-off score ≤ 6/7 on the DFR score showed sensitivity, 80%; specificity, 70% (AUC = 0.760; 95% confidence interval 0.647–0.874, *p* < 0.005). The DTR score showed the best discriminative properties to differentiate PD-MCI from PD-NC using a cut-off ≤ 12/13 points, achieving sensitivity, 86%; specificity, 81% (AUC = 0.870; 95% confidence interval 0.804–0.936, *p* < 0.001). Using crosstabulation, we found DTR scores identified PD-MCI patients with 80% accuracy (see Figure 1).

### Neuroimaging Data

**Figure 2:**
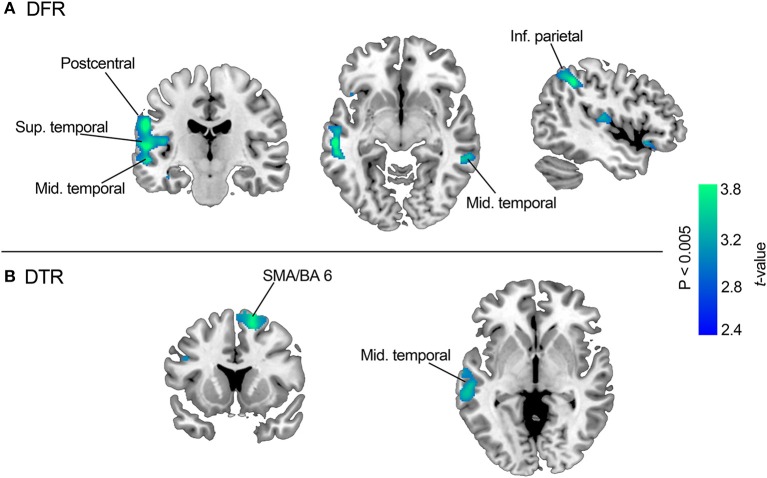
Results of the VBM analysis. The slices show regions with significant GMV decreases in association with poorer FCSRT performance. Section **(A)** (top) depicts GMV associated with DFR performance; section **(B)** (bottom) depicts GMV associated with DTR performance. For depiction purposes results are showed with a *p* < 0.005 (uncorrected) and *k* = 100.

Voxel-wise multiple regression analysis between GMV and DFR and DTR respectively showed significant positive associations between FCSRT performance and GMV in multiple fronto-temporal and parietal areas. Specifically, poorer DFR scores were associated with decreased GMV in the left mid temporal gyrus (BA 21), the paracentral gyrus and the superior temporal gyrus (BA 41) (FWE corrected *p* < 0.05). Less restrictive criteria (uncorrected *p* < 0.005; *k* = 100) showed positive associations with the right mid temporal gyrus, the right superior frontal gyrus and the left inferior parietal gyrus (BA 40). Performance in the DTR was significantly associated with GMV in the right supplementary motor area (SMA), in the right superior frontal gyrus (BA 6) (FWE corrected *p* < 0.05) and in the left mid temporal gyrus (BA 21) (see Table 4, Figure 2 and Supplementary Table 1).

**Table 4:** Results of the GMV voxel-based morphometry and MD-DTI analysis.

**Anatomical region**	**Cluster size**	***T* value**	**MNI coordinates (*x, y, z*)**
**VOXEL-BASED MORPHOMETRY ANALYSIS OF GMV**			
**FCSRT delayed free recall**			
Left mid temporal (BA 21) / postcentral / superior temporal (BA 41)^*^	2,799	4.63	−60, −26, −5 −57, −21, 26 −57, −18, 8
Right mid temporal	551	3.25	60, −39, 9
Left inferior parietal (BA 40)	498	3.76	−47, −45, 45
Right superior frontal	371	3.55	21, 6, 59
**FCSRT delayed total recall**			
Right SMA / superior frontal (BA 6)^*^	1,832	4.44	17, 11, 66 21, 8, 60
Left mid temporal (BA 21)	606	3.90	−60, −26, −5
**MD-DTI ANALYSIS**			
**FCSRT delayed free recall**			
Left superior temporal (BA 22)	1,212	3.72	−46, −7, 1
Left inferior frontal (BA 47)	916	3.44	−40, 14, −5
Left hippocampus	884	4.41	−36, −21, −15
Left mid temporal	509	3.69	−56, −25, −5
Left middle temporal	246	4.23	−54, −59, 1
**FCSRT delayed total recall**			
Left inferior frontal^*^	4,158	4.04	−45, 12, 22
Left postcentral	2,307	3.70	−58, −11, 27
Left superior temporal	1,210	3.31	−62, −16, 1
Left inferior temporal	574	3.79	−40, −18, −19
Left superior temporal pole	553	4.12	−43, 13, −23

* *Cluster level FWE corrected (p < 0.05)*.

**Figure 3:**
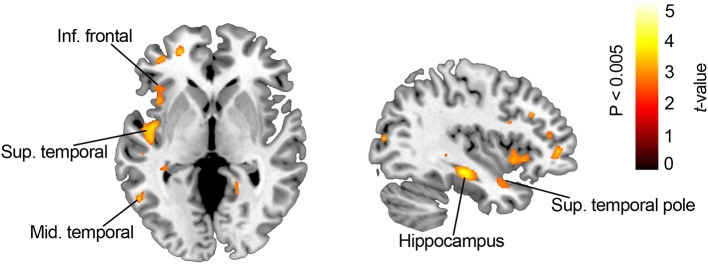
Results of the voxelwise MD analysis. The slices show regions with significant MD increases in association with poorer FCSRT performance. For depiction purposes results are showed with a *p* < 0.005 (uncorrected) and *k* = 100.

Voxel-wise MD multiple regression analysis with DFR and DTR showed a significant association between performance and MD in multiple fronto-temporal and parietal clusters predominantly located in the left hemisphere. Specifically, associations were found between worse DFR and increased MD in the left superior temporal gyrus (BA 22), the left inferior frontal gyrus (BA 47), the left hippocampus, the left mid temporal and the left middle temporal gyrus. Performance in the DTR was significantly associated with MD in the left inferior frontal (FWE corrected *p* < 0.05), the left postcentral, the left superior temporal gyrus, the left superior temporal pole, and the left inferior temporal gyrus (see Table 4 and Figure 3).

Voxel-wise multiple regression analysis between GMV and MD with both IFR and ITR scores did not show any significant association surviving FWE correction.

## Discussion

In the present study we assessed performance in the FCSRT in non-demented PD and compared the performance in this task between PD-MCI and PD-NC. We also explored the discriminative properties of different subscores of the FCSRT and, in a subset of patients; we addressed the structural neuroimaging correlates of FCSRT performance by means of GMV-VBM and MD.

Our results show that, among other neuropsychological measures, PD-MCI patients perform significantly worse than PD-NC and healthy controls in the FCSRT. No significant differences were seen between PD-NC and healthy controls in this respect.

The FCSRT performance in the PD-MCI group was worse than that in PD-NC patients and controls in all the free and cued immediate and delayed recall conditions, suggesting difficulties at level of encoding, consolidation and retrieval. Interestingly, multiple measures of memory performance and specifically a single measure of episodic memory (DTR) correctly classified as PD-MCI up to 80% of the cases. These results emphasize the notion that beyond the prototypical frontal-executive deficits characterizing cognitive impairment in PD, amnestic difficulties are also inherent features of the cognitive changes observed in PD. Accordingly, this indicates that early to mid in the course of PD-MCI, not just frontal-executive, but also amnestic difficulties are present.

Neuroimaging data showed an association between widespread cortical (temporal, parietal and prefrontal) areas and FCSRT performance. Less GMV in mid temporal (BA 21), the superior temporal, the supramarginal, the inferior parietal and the superior frontal gyrus was clearly associated with delayed free recall. Conversely, a more selective involvement of superior frontal regions was found for delayed total recall. All these areas have complex connections that can be grouped into (a) temporal and parietal areas more specialized in storage processes, and (b) prefrontal areas more specialized in retrieval processes (5, 24). Altogether, our results suggest that episodic memory deficits in PD-MCI are sub-served by dysfunction of parieto-temporal and prefrontal-related encoding, consolidation and retrieval processes. Similarly, MD-DTI analyses delineated the involvement of a set of fronto-temporal regions including the temporal pole and the hippocampus. All these regions are connected through the parahippocampal cingulum bundle, which extends along the parahippocampal gyrus, running from the anteromedial temporal lobe to the inferior parietal and occipital lobes (25). The parahippocampal cingulum bundle is closely linked to learning and episodic memory (26–28). Furthermore, microstructural white matter changes in this region have been consistently associated with episodic and recognition memory deficits in amnestic MCI and early AD patients (29–31).

In the only previous study that has analyzed the performance of FCSRT in PD patients with amnestic mild cognitive impairment, PD patients performed worse than healthy controls on delayed free recall, but no differences were found regarding delayed cued recall scores (10). This discrepancy could be explained by both the smaller sample size and the lack of comparison between PD patients with and without MCI, as the study focused on comparing amnestic mild cognitive impairment with healthy controls and patients with amnestic mild cognitive impairment without PD. Although memory deficits in PD-MCI patients are widely considered to be caused by retrieval problems, studies using comprehensive neuropsychological batteries have shown that memory impairment in pre-dementia stages is also the consequence of encoding and storage failure (32). The ability of a memory test, such as the FCSRT, to assess both encoding and retrieval deficits would explain its appropriateness for screening PD-MCI accurately.

Recent studies in newly diagnosed and non-demented PD patients have also underlined the relevance and early development of cortical gray matter changes and DTI-MD alterations in hippocampal and parahippocampal structures as predictors of worsening cognition (33, 34).

This study has several limitations. First, there were fewer PD-MCI patients than PD-NC patients. However, the prevalence of PD-MCI is representative of the one observed in PD patients in the early to mid-stages of the disease. Second, we did not include in the study the measure “trial 1 free recall of the FCSRT” which would have given us more information about encoding. Third, the fact that we did not include patients with dementia limits our ability to see how this population performs on FCSRT. Fourth, not all the participants underwent neuroimaging, and although the number of PD patients with available neuroimaging was comparable to other VBM and DTI studies, imaging data was lacking for the healthy control group used in this study. And fifth, only a small subset of the clusters described in the neuroimaging analyses survived FWE correction.

To our knowledge, this is the first study to look for FCSRT cut-off scores in the screening of PD-MCI by using currently accepted MDS-TF criteria, providing evidence that this test is highly accurate for this purpose. Furthermore, we observed that FCSRT impairment correlates with structural changes in crucial areas of the semantic network and memory storage. The combination of these clinical and imaging findings supports the use of this test as an appropriate neuropsychological tool to detect PD-MCI patients with widespread cortical alterations.

## Data Availability Statement

The datasets generated for this study are available on request to the corresponding author.

## Ethics Statement

The studies involving human participants were reviewed and approved by Comité etico de Investigación Clinica—Sant Pau. The patients/participants provided their written informed consent to participate in this study.

## Author Contributions

AH-B, JP, SM-H, and JK conception, design, data collection, data analysis, data interpretation, writing, and editing. JM-L, FS, RF-B, MB, HB-K, IA-B, JP-P, BP-S, AC, CI, and BG-A data collection and editing.

## Conflict of Interest

The authors declare that the research was conducted in the absence of any commercial or financial relationships that could be construed as a potential conflict of interest.
