# Supplementary material for: The Free and Cued Selective Reminding Test in Parkinson's Disease Mild Cognitive Impairment: Discriminative Accuracy and Neural Correlates
Source: Front Neurol. 2020 Apr 21;11:240. doi: 10.3389/fneur.2020.00240 (PMC7186438; doi:10.3389/fneur.2020.00240)
Supplement: Supplementary file 1 [file Table_1.docx]

**Supplementary Tables:**

**Supplementary Table 1:** Clinical and sociodemographic characteristics of PD patients with MRI acquisition.

|  | PD-Total Sample  (n=56) | | PD-MCI  (n=10) | | PD-NC  (n=46) | | *Sig.^a^* |
| --- | --- | --- | --- | --- | --- | --- | --- |
|  | M | SD | M | SD | M | SD |  |
| **Age**, yearsAadax Span 3374.EDU BARCELONA AAA | 69.2 | 6.8 | 73.9 | 5.5 | 68.5 | 6.9 | 0.033 |
| **Education**, years | 12.4 | 4.5 | 9.0 | 4.4 | 13.1 | 4.2 | 0.012 |
| **Gender**, % males | 36 | 64% | 4 | 40% | 29 | 63% | 0.057 |
| **PD symptoms duration**, years | 5.9 | 3.4 | 6.2 | 3.7 | 5.9 | 3.4 | 0.773 |
| **UPDRS-III** | 24.8 | 8.4 | 29.2 | 7.0 | 24.4 | 8.5 | 0.122 |
| **Hoehn/Yahr stage** | 2.0 | 0.4 | 2.3 | 0.2 | 2.0 | 0.4 | 0.021 |
| **Schwab & England** | 87.4 | 6.4 | 83.3 | 5.0 | 88.2 | 5.7 | 0.160 |
| **Medication**, mg LEDD | 632.8 | 342.4 | 652.4 | 363.3 | 619.6 | 354.0 | 0.802 |
